# Country experiences on the path to exclusive use of validated automated blood pressure measuring devices within the HEARTS in the Americas Initiative

**DOI:** 10.1038/s41371-022-00706-9

**Published:** 2022-07-11

**Authors:** Cintia Lombardi, Dean S. Picone, James E. Sharman, Norm R. C. Campbell, Rafael Farias, Stephanie Guerre, Anselm Gittens, Melanie Paccot, Nilda Villacres, Yamile Valdes, Pedro Ordunez

**Affiliations:** 1 Department of Non-Communicable Diseases and Mental Health, Pan American Health Organization, Washington, DC USA; 2 Menzies Institute for Medical Research, University of Tasmania, Hobart, TAS Australia; 3 Departments of Medicine, Physiology and Pharmacology and Community Health Sciences, Libin Cardiovascular Institute of Alberta, Calgary, AB Canada; 4 National Institute of Metrology, Quality and Technology (INMETRO), Sao Paulo, Brazil; 5 Pan American Health Organization, Santo Domingo, Dominican Republic; 6 Saint Lucia Bureau of Standards, Castries, Saint Lucia; 7 Department of Non-Communicable Diseases, Ministry of Health, Santiago, Chile; 8 Pan American Health Organization, Quito, Ecuador; 9 University Hospital General Calixto Garcia, Havana, Cuba

**Keywords:** Diagnosis, Disease prevention

## Abstract

The aim of the HEARTS in the Americas initiative is to promote the adoption of global best practices in the prevention and control of cardiovascular diseases, and improve the control of hypertension. HEARTS is being implemented in 21 countries and a diverse set of actions and measures are in progress to improve exclusive access in primary health care facilities to automated blood pressure measuring devices that have been validated for accuracy. The purpose of this manuscript is to illustrate these efforts, mainly in the regulatory and public procurement arena, and to present information on common challenges and solutions identified. Examples from six countries confirm the need for not only a robust regulatory framework to increase availability of validated automated blood pressure measuring devices but also a comprehensive strategic approach that involves relevant stakeholders, includes a multi-pronged approach and is associated with a national program to prevent and control non communicable diseases.

## Introduction

High blood pressure (BP) is a leading risk factor for cardiovascular disease, which is a major health burden on the Region of the Americas [1]. HEARTS in the Americas, the regional adaptation of WHO Global HEARTS initiative [2], aims to promote and implement global best practices in the prevention and control of hypertension and cardiovascular diseases risk management, and one key pillar of the initiative is the accurate measurement of BP [3]. A critical step in this strategic effort is to ensure the exclusive use of accuracy validated automated blood pressure measuring devices (BPMDs) in primary health care (PHC) settings by 2025. This goal is crucial because BP measurements are more accurate using validated automated BPMDs, compared with non-validated automated BPMDs [4–6]. Other critical goals of the BP measurement pillar are adoption of a correct measurement protocol and training of personnel [7, 8]. Accuracy validation refers to the rigorous clincal testing of BPMDs against a gold standard to determine accuracy using a scientifically validation protocol. Further information can be found elsewhere in this special issue [9].

In work by PAHO and countries implementing HEARTS, regulatory deficiencies have been identified that allow automated BPMDs to be marketed without evidence of accuracy validation [10], which is similar to the situation in most countries globally [11, 12]. The regulatory focus lies mostly on the safety and other quality assurance aspects of automated BPMDs instead of assurance of accuracy. Only one country in the region of the Americas implementing HEARTS does require evidence of accuracy validation for approval of automated BPMDs, however it also accepts BPMDs approved by other regulatory agencies that do not require evidence of validation. Therefore, although the proportion of validated automated BPMDs available in the Americas is unknown, it is likely that most have not undergone accuracy validation testing.

Since 2017 the number of countries implementing HEARTS in the Americas has grown, and there are currently 21 countries enrolled in the initiative at different stages of implementation and level of expansion. The efforts to improve access to validated automated BPMDs are different for each country, in line with its priorities, health system maturity including regulatory, economic, and political conditions. Nevertheless, there are common themes between countries for successes, challenges and the steps taken improve access to validated automated BPMDs. The purpose of this manuscript is to provide examples from six selected countries that are trying to address the problem of non-validated automated BPMDs. The information contained in this manuscript may be useful for other countries that are considering changes to regulatory and procurement processes with the goal of achieving exclusive use of automated BPMDs clinically validated for accuracy.

In order to assist the transition towards exclusive use of validated automated BPMDs, and in line with the WHO recommendations [13], the Pan American Health Organization (PAHO) provides technical cooperation to the countries implementing HEARTS in various ways [3]. Recent efforts include regional and bilateral meetings conducted with representatives of Ministries of Heath and National Regulatory Agencies in November and December 2020 to explore strategic and feasible actions and identify a timeline for each country. In August 2021, PAHO led a virtual consultative technical meeting about recommendations for regulatory frameworks related to automated BPMDs. During the technical meeting there were expert presentations and group discussions regarding the draft recommendations for regulatory frameworks, including foreseeable challenges and pathways to implementation. The resulting document [14] is a tool to be used by Ministries of Health and national regulatory authorities to develop, implement and enforce regulations for the exclusive pre-market approval of validated automated BPMDs. During this process, a strategy was developed to systematize the work being conducted at the country level, with the technical cooperation by PAHO, to achieve the goal of exclusive use of validated automated BPMDs, which is comprised by several elements [15], as seen in Box 1.

Any modifications aiming to improve regulations or procurement mechanisms related to automated BPMDs need to be coordinated, progressive and sustainable and therefore must be integrated into a national health strategy and cardiovascular health programs. Policies should also be flexible enough to account for technological innovations and advancement of research methods.

Box 1 Elements of HEARTS in the Americas strategy to ensure exclusive use of validated automated blood pressure measuring devices (BPMDs) in primary health care facilities

- Understanding the regulatory context through mapping of regulatory bodies and processes.
- Creating awareness by bringing the concept and know-how of accuracy validation into the public health agenda.
- Training on protocols for accuracy validation of automated BPMDs to enable countries to perform studies and educate on the difference between accuracy validation and other mechanisms of quality assurance.
- Providing information on which automated BPMDs are validated and available in the Region of the Americas for acquisition by the public sector.
- Developing technical specifications for procurement of automated BPMDs to be used as criteria for procurement and for regulations.
- Guidance and technical support in the development, implementation and monitoring of regulations.
- Promotion of a process that counts on the ample participation of key stakeholders and that is implemented gradually.

## Examples of approaches to improve access to validated automated BPMDs in the Americas at the country level

Different approaches to improve access to validated automated BPMDs have been taken by governments of each country, based on their priorities, regulatory systems, political and economic characteristics. Examples of key actions and challenges specific to Brazil, Chile, Cuba, Dominican Republic, Ecuador and Saint Lucia were provided by representatives of governments (Ministry of Health and/or regulatory authorities) of selected HEARTS countries. The narratives provided were checked against other information previously obtained and online records for consistency, condensed and edited for clarity and validated by the coauthor representatives (RF, MP, YV, NV, SG, AG). An overview of the common strengths, challenges, key actions and lessons are summarized in Table 1.

**Table 1:** Summary of strengths, challenges and key actions in six countries relating to improving access to validated automated blood pressure measuring devices (BPMDs).

Country	Focus	Strengths and challenges	Lessons to other countries
Brazil	Regulations	Strength: Strong regulatory system, with high technical capacity. Challenges: Compliance and enforcement. Approval of non-validated automated BPMDs of approved by authorities of IMDRF participating countries. Key action: Inclusion of ISO 81060-2:2018 and other accuracy validation standards on automated BPMDs marketing registration requirements.	Regulate acceptance of approval by other countries, including those belonging to the IMDRF. Enforce regulations, including the use of post-market surveillance
Chile	Procurement	Strength: Strong health care and health information system. Challenges: Regulatory bottleneck. Decentralized health system. Key actions: Procurement of a substantial number of validated automated BPMDs for use in PHC. Promotion of accuracy validation through issuing of protocols and recommendations by the MoH.	Ensure that procurement mechanisms are normative and enforceable. Follow regulatory pathway at the same time.
Cuba	Regulations Clinical validation studies	Strength: Domestic manufacturing and technical support to perform accuracy validation studies. Challenge: Budgetary limitations. Regulatory bottleneck. Key action: Performing accuracy validation study of a domestically produced automated BPMD.	Streamline regulatory processes. Obtain technical cooperation to build capacity to perform accuracy validation studies
Dominican Republic	Procurement	Strength: Political will and commitment to ensure exclusive use of validated BPMDs in PHC. Challenge: Resistance to the use of validated, semi-automated BPMDs by the medical profession. Key action: Acquisition of substantial number of validated automated BPMDs for use in PHC.	Foster awareness and knowledge among the medical profession of the importance of using automated or semi-automated BPMDs that have been validated for accuracy.
Ecuador	Procurement	Strengths: Commitment by the MoH to procure automated BPMDs that have been validated for accuracy. Capacity in BP measurement strengthened trough virtual courses. Challenge: Budgetary limitations constraining the purchase of new automated BPMDs Key action: Development of technical specifications for procurement of automated BPMDs.	Strengthen procurement mechanisms through adoption of technical specifications that include accuracy validation to circumvent regulatory bottleneck.
Saint Lucia	Procurement and regulations	Strength: Small population size and flexible administrative structure makes it possible to adopt many creative measures. Challenge: Regulatory changes requiring extensive consultation and therefore time. Key action: Promotion of validated automated BPMDs at the population level using social media.	Find creative ways that respond to reality of the country, including the use of social media and modification of services provided by government. Adopt solutions that take advantage of both strengths and weaknesses.

*BP* blood pressure, *BPMD* blood pressure measuring device, *IMDRF* International Medical Devices Regulations Forum, *MoH* Ministry of Health, *PHC* primary health care.

### Brazil is setting procurement goals for validated BPMDs

Accuracy validation based on ISO 81060-2: 2018 or other validation protocols is currently required for the marketing of automated BPMDs in Brazil. Manufacturers (or importers) must submit a clinical investigation report to the National Institute of Metrology (INMETRO), which analyzes the report content to confirm that all points required by the protocols are met. At INMETRO, investigation reports are reviewed by metrology researchers, generally with a background in engineering and experience in metrology, focusing on healthcare.

The accuracy validation study results are provided directly by the manufacturer. However, the results are not required to be included on registries of validated automated BPMDs or published in peer-reviewed scientific journals. This suggests a lack of independence of the manufacturer from the accuracy validation data, which is not recommended as best practice [11, 16]. For this reason, INMETRO have been considering a revision of its regulation to include requirements that ensure that independent clinical investigations are carried out and compliance with protocol requirements. Another modification under consideration would limit equivalence statements between models and set parameters for proof of equivalence. These revisions are expected to take place in 2022-2023. The decisions to make the above modifications were made as a result of recommendations from the HEARTS in the Americas initiative. Finally, despite having a substantial capacity, a robust regulatory and surveillance system established customs control, and post-market surveillance mechanisms, actions are limited in Brazil due to the lack of human and material resources needed to enforce actions in a country with continental dimensions.

### In Chile, HEARTS implementation resulted in a change in the purchase pattern

The implementation of the HEARTS Initiative in 2017, in which Chile was part of the first cohort, has reinforced the need to improve the quality of BP measurement. Since 2012, the purchase of validated automated BPMDs was recommended by the Ministry of Health (MoH). This recommendation was stressed in 2018 by HEARTS implementation with the development of the BP measurement protocol, videos of correct BP measurement and educational materials for patients, all emphazing that automated BPMDs must be validated for accuracy. The MoH’s National Supply Center reported that between 2018 and 2020, automated BPMD purchases increased from 15% to 75% of the total number of devices. Likewise, the proportion of validated automated BPMDs acquired for use in the public sector increased from 34% in 2018 to 64% in 2020. It is important to note that there are other purchasing mechanisms, such as those defined by the different Health Services (decentralized units of the National Health System), which are autonomous to make their own decisions.

In 2021, a BP measurement protocol was published on the MoH website stressing the recommendation of only using validated automated BPMDs [17]. In addition, the MoH also published a memo with recommendations for use and acquisition of validated BPMDs which was sent to the directors of all health services areas in the country and several units within the MoH [18].

Chile still has challenges from the regulatory perspective because the measurement protocol and the recommendations for acquisition of automated BPMDs aforementioned are not normative yet and thus are not enforceable. To address this challenge, a group including representatives of government bodies responsible for the purchase and surveillance of medical devices across Chile is currently working on a regulation proposal that includes impact assessment and identification of the automated BPMDs available in all health care facilities across the country.

### Cuba is conducting an accuracy validation study of a locally produced BPMD

Cuba cannot access validated automated BPMDs available in the global market due to their high cost and other economic and trade restrictions. Therefore, the validated automated BPMDs available as of December 2021 were obtained mainly through international technical cooperation projects. However, a Cuban medical equipment manufacturer has developed automated BPMDs for 24-hour ambulatory monitoring and, as of 2016, an automated BPMD for home and office use. The locally produced automated BPMDs are currently used at all levels of care. In 2018, due to the HEARTS Initiative emphasis on replacing aneroid devices, domestic automated BPMDs were introduced and rapidly expanded to provide at least one device for approximately 70% of the 11,000 PHC teams in the 489 health centers across the country. However, those devices are not yet clinically validated using the ISO 81060-2:2018 standard or any other validation protocol.

Catalyzed by the HEARTS in the Americas, in 2019, training on accuracy validation studies was provided by experts from the University of Alberta, Canada. The purpose of the training was to develop the technical expertise and knowledge to conduct validation studies according to the highest international standards and assess the accuracy of the locally produced automated BPMDs. Subsequently, the National Hypertension Commission, an independent technical body, approved the formation of a technical group to conduct an accuracy validation study under the academic coordination of the Institute of Cardiology and Cardiovascular Surgery, with the participation of the State Center for the Control of Medicines, Equipment and Devices, a WHO collaborating center, to verify the accuracy of locally produced automated BPMDs. There was no industry participation in any step of the process.

In parallel to the accuracy validation effort, the conditions will be created to modify the regulatory framework to incorporate ISO 81060-2:2018 into Cuban Standards to ensure exclusive use of validated automated BPMDs before 2025. Finally, the availability of automated BPMDs must be improved, including expanding the production of the current model and the development and commercialization of new ones, with more capabilities, such as data transmission features.

### The Dominican Republic is using validated semi-automated BPMDs in PHC facilities but finding resistance from health professionals

A challenge in the Dominican Republic is the precariousness of the electrical power supply in many remote health centers and the absence of uninterrupted availability of batteries to operate automated BPMDs. For these reasons, the HEARTS national managing team chose to temporarily maintain the use of aneroid BPMDs in some facilities but also to provide additional training for health personnel focused on the correct use of automated BPMDs. Simultaneously, an exhaustive search was conducted to identify automated or semi-automated BPMDs that would not need batteries to operate. The search led to choosing a validated semi-automatic BPMD with rechargeable batteries that is accurate in pregnancy and meets the WHO criteria for use in a low-resource setting [19]. This device uses a traffic light system associated with the reading of BP figures. Sixty devices were acquired with donated funds and were designated for use in PHC facilities. An additional factor in the choice of this device was the high maternal mortality rate due to eclampsia in the country, which means that accurate BP measurements in pregnancy are crucial.

Since this was the first time a digital device would be used in the country, a 15-day study was conducted with a small sample of health personnel to explore how they would respond to this change. This study, which is yet to be published, was conducted by taking measurements alternatively with aneroid devices and the semi-automated BPMDs on the same patient. There was some resistance to change by the medical profession. To overcome this resistance progressively, health care personnel are being trained through local HEARTS workshops and the HEARTS BP measurement course, to educate them on the difficulty of accurately diagnosing hypertension with an aneroid sphygmomanometer.

### In Ecuador, the Ministry of Health is setting a regulation to procure validated BPMDs for use in public facilities

In 2018, 94 validated automated BPMDs were acquired to conduct the Ecuador non-communicable diseases risk factors survey and PHC facilities that had started implementing HEARTS. Unfortunately, budget cuts within the Ecuadorean MoH have since hindered the purchase of new validated automated BPMDs. However, the initial devices were followed by the acquisition of an additional 468 validated automated BPMDs, including 300 devices through liaison with the HEARTS coordinating team and international cooperation agencies. As of December 2021, 352 health centers were implementing HEARTS, which corresponds to 18% of the country’s 1940 health centers. Approximately 85% of these 352 centers have validated automated BPMDs, which means 15% of the total in the country.

In addition, the HEARTS in the Americas pillar on education and training has found great receptivity demonstrated by the high number of health professionals taking the HEARTS virtual course on BP measurement [8]. The nurses of all health centers implementing HEARTS have been trained to train the other team members on the correct BP measurement and use of validated automated BPMDs during demonstrative sessions, which occurred in approximately 80% of the health centers implementing HEARTS.

To ensure exclusive public procurement of validated automated BPMDs for HEARTS implementing centers, technical specifications that include international accuracy validation standards have been developed by the MoH with the support of. In addition, the process to make the standards enforceable for the acquisition of BPMDs for use in any public facility, especially those in PHCs, has been initiated within the MoH and is expected to be approved in the first trimester of 2022.

A challenge has been the limited availability of validated automated BPMDs in the national market. This was identified when procuring BPMDs for use in HEARTS implementing centers in 2021, by analysis of the National Agency for Regulation, Control and Sanitary Surveillance (ARCSA) database of BPMDs with pre-market approval for sale. Only one had presented proof of passing accuracy validation testing from the five models registered for sale. An additional challenge is the lack of knowledge of the advantages of using automated BPMDs and preventive maintenance to extend their useful life. Provision of information and advocacy with health care personnel and managers are being conducted to address these issues. However, a key step will be modifying regulations to include accuracy validation as a requirement for registration at ARCSA, which is underway.

### Saint Lucia is adopting multiple approaches to promote accuracy validation of automated BPMDs

In Saint Lucia, registration of automated BPMDs for sale in the country is dependent upon approval by the Saint Lucia Bureau of Standards (SLBS), which has responsibility for enforcement of the Metrology Act. The Act is a law which gives the SLBS responsibility for approval and verification of measuring instruments used in the field of public health, including automated BPMDs. However, achieving the goal of enacting legislation that would ensure the exclusive sale of accuracy validated automated BPMDs in the country involves other government sectors, and, to that end, discussions between the stakeholders, such as MoH, Ministry of Commerce, Attorney General’s Chambers, and Customs, have been underway.

Meanwhile, in May 2021, accuracy validation became a prerequisite for verification of accuracy, performance, and calibration by the SLBS, as per OIML R149 requirements. A campaign to promote the use of validated BPMDs has been carried out with the use of social media, and two webinar presentations were given by the SLBS to the medical sector and importers recommending that only validated automated BPMDs approved for sale in the country and tested for accuracy against established protocols be used. One large pharmacy chain in Saint Lucia imported a stock of validated automated BPMDs for sale to the public as a result of an SLBS presentation to the medical sector and it is anticipated that competitive forces will play a role in ensuring that other suppliers follow suit. The public service announcement from the SLBS on the verification of automated BPMDs, which first aired in 2017, and is presently on the SLBS’ YouTube channel will be edited to include specific information on validated automated BPMDs.

Among the challenges are online purchases of non-validated automated BPMDs at distributors and retail outside Saint Lucia, pressure from suppliers to sell the remaining stock of non-validated automated BPMDs and the variability of information available on online registries.

### Summary of achievements by countries to date

The six countries that were the focus of this manuscript have shown the political will to reach the exclusive availability of validated automated BPMDs. Indeed, most of the facilities implementing HEARTS began to use accuracy-validated automated BPMDs, and this represented an important stimulus to advance this strategic work. The general achievements by these six countries showed in concrete actions are twofold: (a) sectors of governments have been investing politically and administratively in modifications to regulations that do not require a substantive and complex legislative process; and (b) in parallel, improved procurement toward exclusive access of validated automated BPMDs has been at least partially achieved. The improvement of procurement mechanisms has focused on defining technical specifications that include accuracy validation. In addition, lack of access to validated automated BPMDs has been counteracted by local production of devices and cooperative procurement approaches. The lessons shared in Table 1 may be of benefit to other countries seeking to achieve exclusive use of validated BPMDs.

### Summary of ongoing challenges

There are many ongoing challenges confronted by countries implementing HEARTS. The common challenges are related to changing regulations and/or legislation, budgetary limitations and resistance to uptake of automatic BPMDs by health professionals, particularly physicians (Table 1). Steps to overcome the regulatory and legislative challenges vary between countries but consist of efforts to strengthen the regulatory processes to include accuracy validation on existing or new legally binding measures such as decrees and regulations, update medical devices laws if needed, define enforcing mechanisms, and emit public procurement directives. The budgetary limitations that prevent the purchase of validated BPMDs should be addressed progressively by promoting centralized procurement and using a regional cooperation mechanism such as the PAHO Strategic Fund [20]. Resistance to uptake of automated BPMDs is a concerning challenge. As seen in other areas of health care, adoption of innovation is not a process that occurs easily. There is a need to understand the individual profiles and motivation to change by health professionals in each country [21], and they need to be informed about the major features and benefits of the innovation [22]. In this case, they are a more accurate diagnosis of high BP and reduced time-burden because other team members can perform BP measurements.

### Next steps toward achieving exclusive use of validated BPMDs in the Region of the Americas

The key next step for countries is to develop, implement and enforce regulations that guarantee that only those automated BPMDs that have passed an accuracy validation study are approved for sale in the country. This step will require the participation of different actors. It may need to start with the education and advocacy actions, providing information on the benefits of using validated automated BPMDs to policymakers, procurement officers, health care providers and managers of health services. In addition, advocacy involving medical and professional societies, patients (or consumers) groups and civil society may create pressure to improve the quality of care. These processes, which are underway to different degrees in each country, may require understanding the audience, timing, and engaging with real-world policy-making [23].

Another next step that will improve access to and knowledge of validated automated BPMDs is the development of country-specific lists or a regional list of validated automated BPMDs relevant to the Region of the Americas. A key feature of this list, in addition to the standard specifications of devices, should be information about the power source of the validated automated BPMDs, because access to reliable power varies greatly across the Americas. There has been a substantial increase of awareness of the importance of using accuracy validated automated BPMDs in countries implementing HEARTS. However, there are still several gaps in knowledge of technical aspects of automated BPMDs, which need to be summarized and disseminated to procurement officers.

## Conclusion

The challenges that the countries face to reach the goal of exclusive use of validated BPMDs in PHC are enormous and multifactorial. This confirms the need for a robust regulatory framework and comprehensive strategic approach to exclusive use of clinically validated automated BPMDs at regional level. HEARTS in the Americas has adopted a strategy to overcome these challenges over time, concomitantly conducting parallel efforts in several areas. The successful implementation of the initiative depends on a coordinated effort across government and other stakeholders that will contribute to better diagnosis and management of hypertension in the Americas.

## Summary

### What is known about topic

- Blood pressure measuring devices that have been clinically validated have shown to be more accurate than those that have not.
- Most blood pressure measuring devices found in the global market have not been clinically validated.
- The regulatory framework pertinent to the marketing of blood pressure measuring devices in the Region of the Americas is weak and fragmented. There is a need to implement regulations and procurement mechanisms to ensure exclusive use of validated measuring devices.

### What this study adds

- There are multiple strategies to reach the goal of exclusive use of validated automated blood pressure measuring devices, and these should be adopted concomitantly to the extent of their feasibility.
- The diversity of challenges faced by countries are related to their political and economic landscape, health systems and regulatory maturity, and factors such as geography and population size.
- The different approaches to these challenges describe in the paper could be used as lessons by other countries in their efforts to reach the goal of exclusive use of validated automated blood pressure measuring devices in primary health care.
